# Developing a Logic Model Framework for Community-Dwelling, Non-Familial Intergenerational Programmes

**DOI:** 10.1007/s10935-025-00880-9

**Published:** 2025-12-06

**Authors:** Ludwig Grillich, Karolina Seidl, Alexander Pell, Jana Nikitin, Anton-Rupert Laireiter

**Affiliations:** 1 Department for Evidence-Based Medicine and Evaluation, University for Continuing Education Krems, Dr. Karl Dorrek-Straße 30, 3500 Krems an der Donau, Austria; 2 Department of Clinical and Health Psychology, Faculty of Psychology, University of Vienna, Vienna, Austria; 3 Department of Developmental and Educational Psychology, Faculty of Psychology, University of Vienna, Vienna, Austria; 4 Department of Psychology, University of Salzburg, Salzburg, Austria

**Keywords:** Intergenerational programmes, Programme implementation, Healthy ageing, Social isolation, Loneliness, Community engagement, Qualitative research, Logic model

## Abstract

Community-dwelling, non-familial intergenerational programmes (IPs) have emerged as a promising approach to preventing social isolation and loneliness among older adults. However, there is a need for a comprehensive understanding of the key components, underlying mechanisms, and contextual factors involved in implementing such programmes. This study aimed to develop a comprehensive logic model framework for non-familial IPs to guide future programme design and implementation. A qualitative approach was employed to analyse the ‘Post für Dich!’ [Mail for You] programme in rural Lower Austria through semi-structured interviews with older adults, implementation agents, and community leaders, along with a focus group of primary school children, and a document analysis. The resulting logic model framework identifies critical inputs (e.g., local coordinator, engaged implementation agents, meeting spaces, programme flexibility), highlights the role of existing organisational settings in facilitating access to target groups, and outlines key activities (programme initiation, coordination, letter exchanges, and intergenerational events). It reports multifaceted benefits for participants, such as improved attitudes, dispelled age stereotypes, expanded social networks, and an enriched daily life for older adults. Potential community-level impacts, such as fostering multigenerational awareness and integration, are noted. This empirically grounded framework serves as a practical tool for systematically designing, implementing, and evaluating IPs across diverse settings. It can facilitate knowledge transfer, evidence-informed practice, and capacity-building efforts, contributing to the effective implementation of these interventions.

**Supplementary Information:**

The online version contains supplementary material available at 10.1007/s10935-025-00880-9.

The global population is ageing rapidly, with a projected increase in the number of individuals aged 65 and older from 703 million in 2019 to 1.5 billion by 2050 (United Nations Department of Economic Social Affairs, 2020). This demographic shift has led to a growing concern about loneliness among older adults, which can have detrimental effects on their physical and mental well-being (Krzeczkowska et al., 2021).

Loneliness, a distressful subjective experience, occurs when one’s social needs are not met by the quality or quantity of current relationships (Hawkley & Cacioppo, 2010). Intergenerational programmes (IPs) have emerged as a promising approach to counteract loneliness in older adults by facilitating active connections between people of different generations (Peters et al., 2021). These programmes provide new contexts for intergenerational encounters, which have diminished with the decline of multigenerational households and the rise of age-homogeneous neighbourhoods (Stein, 2012).

Research has shown that IPs can improve various outcomes for older adults, such as reducing depression and loneliness and enhancing quality of life (Lee et al., 2020; Murayama et al., 2015, Murayama et al., 2019; Peters et al., 2021). Additionally, IPs have been associated with reduced age stereotyping, improved cognitive function, and better health for older adults, and for children, improved empathy and self-esteem (Giraudeau & Bailly, 2019; Peters et al., 2021; Ronzi et al., 2018). The National Institute for Health and Care Excellence (NICE) specifically recommends intergenerational activities to maintain and improve the mental well-being and independence of people aged 65 and over (NICE, 2015). Despite these documented benefits, organisations face significant challenges in implementing and sustaining effective IPs, including participant recruitment, resource allocation, cross-generational engagement, and impact measurement. A structured framework is needed to address these practical obstacles.

IPs are complex interventions involving several interdependent components that may also interact with characteristics of the target group and the local context (Skivington et al., 2021). The new framework for developing and evaluating complex interventions (update of Medical Research Council [MRC] guidance) emphasises the importance of developing a theoretically robust conceptual framework or causal model that maps out the key components, mechanisms of action, and contextual factors involved (Skivington et al., 2021).

Despite growing recognition of IP’s potential benefits, the literature lacks transparency regarding underlying mechanisms (Laging et al., 2022) and empirical evidence about implementation best practices (Peters et al., 2021). The heterogeneity in programme contexts, sample designs, dosage, and duration necessitate further research to enable wider implementation and generalizability (Krzeczkowska et al., 2021).

In line with MRC guidance (Moore et al., 2015) and the standards of evidence for prevention science (Gottfredson et al., 2015), a clear theory of causal mechanisms, a statement of ‘for whom’ and ‘under what conditions’ the intervention is expected to be effective, and identification of the core components are essential prerequisites for replicating the impact of a complex intervention.

The aim of this study is to develop a comprehensive logic model framework for community-dwelling, non-family IPs. By identifying key inputs, processes, and outcomes, we aim to uncover the mechanisms underlying the successful implementation of these programmes. The proposed framework will serve dual purposes: advancing theoretical understanding of intergenerational engagement and providing a practical tool for programme conceptualisation and refinement.

## Methods

We employed a qualitative approach to develop a logic model based on the ‘Post für Dich!’ case study, following new MRC guidance for developing and evaluating complex interventions (Skivington et al., 2021). The study was approved by the Ethics Committee of the University of Krems, Lower Austria (reference number: EK GZ 50/2021–2024). A logic model is a plausible and reasonable model of how a programme will work under certain environmental conditions to solve identified problems (Bickman, 1987).

### Description of the Case

The ‘Post für Dich!’ (‘Mail for You!’) programme was a non-familial IP implemented in Reichenau an der Rax, a rural village with around 2,500 residents in Lower Austria, from 2016 to 2023. The programme involved older adult residents of the ‘AktiVital’ independent living community and fourth-grade children (ages 9–10) from the local primary school Volksschule Reichenau.

The main objectives of the programme were to:

1. Strengthen social connections between older adults and children.
2. Reduce social isolation and loneliness among older adults.
3. Support literacy skills development in primary school children.
4. Build intergenerational relationships between the two groups.

Key components included the following:

1. Monthly intergenerational letter exchange.
2. In-person reading sessions.
3. Regular intergenerational group activities and excursions.

Materials provided included writing prompts, a video message from the older adults to the children, and children’s literature for the reading component.

The programme was integrated into the school curriculum and led by the director of the independent living community and the classroom teacher, with personnel costs covered by the municipality.

### Data Collection

We conducted seventeen semi-structured interviews, one focus group, and a document analysis between July 2022 and June 2023. Table 1 provides an overview of data collection methods and participants.

**Table 1:** Overview of data collection methods and participants

Method	Participants	Number	Duration
Face-to-face interviews	Older adults (70–85 years)	13	42 min^a^
	Project initiator/manager	1	90 min
Online interviews	Class teacher	1	40 min
	Mayor and deputy mayor	2	40 min
Focus group	Primary school children (10–11 years)	17	45 min
Document analysis	Programme descriptions, activity logs	Not applicable	Not applicable

^a^average time

Recruitment was facilitated by the director of the independent living community and the classroom teacher. All adult participants provided written informed consent. For children, parental consent was obtained, and children provided assent.

Interviews with older adults were conducted in a private room within the independent living community, while the children’s focus group took place in their classroom during a regular school lesson. The teacher and facility director supported the organisation to ensure a familiar and comfortable setting for participants. These familiar environments positively influenced participant engagement and openness during data collection. The interview with the class teacher was conducted in person at the school, while the mayor and vice mayor were interviewed online via video call, providing a flexible yet focused setting for each.

Interview guides were developed based on Witzel’s problem-centred interview approach, tailored to each participant group (Witzel, 2000). The focus group guide followed Przyborski and Wohlrab-Sahr’s (2021) framework. All participants provided written informed consent for audio recordings and data processing (including gender, age range, and occupation). The recordings were transcribed and anonymised before being deleted. Data from children and older adults were analysed in an indirectly person-related manner for additional protection. The class teacher, mayor, and deputy mayor were informed that their distinctive roles might limit full anonymisation despite removing identifying details, and they provided explicit consent. Data were stored securely with access restricted to the research team, ensuring compliance with the General Data Protection Regulation (GDPR). For document analysis (Döring, 2023), we systematically examined programme descriptions and recorded the activities, target groups, and objectives outlined.

### Analytic Strategy

For the analysis process, we followed a hybrid approach, combining deductive and inductive coding within the framework of Bran and Clarke’s (2012) six-step approach for thematic analysis. The hybrid strategy enabled a rigorous yet flexible method for systematically identifying patterns and themes from multiple sources within the qualitative data. Initially, we utilised a deductive coding approach using logic model categories and terminology (McLaughlin & Jordan, 2015) as an initial coding framework. This framework consisted of predefined abstract categories, such as Inputs, Incomes, Activities, Outputs, Outcomes, Impacts, and Context. Prior to data coding, we enhanced this initial framework by integrating two additional theoretical frameworks: the COM-B model of behaviour (Michie et al., 2011) and the four-level taxonomy of outcomes (Kirkpatrick & Kirkpatrick, 2016). This theoretical integration occurred during the framework development phase, before any empirical coding commenced.

While McLaughlin and Jordan (2015) categorise outcomes temporally (short-term, intermediate, long-term), we adopted a theory-based approach, differentiating three outcome types based on the COM-B model and Kirkpatrick´s taxonomy:

• Outcomes I: Encompassing stabilisation or changes in knowledge, attitudes, social values, or skills (aligning with Kirkpatrick’s levels 1 and 2, and the Capability and Motivation components of COM-B).

• Outcomes II: Including stabilisation or changes in behaviour or actions (aligning with Kirkpatrick’s level 3 and the behaviour component of COM-B).

• Outcomes III: Representing stabilisation or changes in the living situation or status of the programme’s target groups (aligning with Kirkpatrick’s level 4).

This theoretically informed subcategorisation of outcomes was established before data analysis began and enhanced our analysis by providing a more detailed framework for understanding the nature and progression of the programme effects. The initial coding framework is presented in Table 2.

**Table 2:** Initial coding framework

Category	Description	Theoretical Foundation
Inputs	Assets, resources, and personnel dedicated to programme operations.	McLaughlin and Jordan (2015)
Incomes	Knowledge, skills, abilities, attitudes, needs, social values, and competencies that beneficiary groups bring to the programme.	McLaughlin and Jordan (2015)
Activities	Actions, processes, and events implemented within the programme.	McLaughlin and Jordan (2015)
Outputs	Direct products, services or deliverables generated by programme activities.	McLaughlin and Jordan (2015)
Outcomes I	Acquisition or modification of knowledge, attitudes, social values, and skills of the programme’s beneficiary groups as a result of participation.	Kirkpatrick levels 1–2; COM-B: Capability & Motivation (Michie et al., 2011; Kirkpatrick & Kirkpatrick, 2016)
Outcomes II	Adoption of new behaviours or modification of existing ones among the programme’s target groups.	Kirkpatrick level 3; COM-B: Behaviour (Michie et al., 2011; Kirkpatrick & Kirkpatrick, 2016)
Outcomes III	Sustainable changes in participant situation or status.	Kirkpatrick level 4 (Kirkpatrick & Kirkpatrick, 2016)
Impacts	Long-term, system-level changes in the community structure, influenced by the programme’s activities.	McLaughlin and Jordan (2015)
Context	Environmental, organisational, and sociocultural factors influencing programme implementation, outcomes, and impacts.	McLaughlin and Jordan (2015)

Within these broad, predetermined deductive categories, we then employed an inductive approach, allowing for the emergence of new codes and themes directly from the data. This inductive process enabled us to capture nuanced aspects of IPs and the specific relational dynamics observed in our case study. All inductive developed codes could be successfully mapped to the predetermined deductive categories, confirming the appropriateness and completeness of our integrated theoretical framework. This hybrid approach aligns with qualitative research methodology that recognises the value of both theory-driven and data-driven analysis in building a comprehensive understanding of complex social phenomena (Fereday & Muir-Cochrane, 2006). In total, 769 text segments were coded across the dataset. The coding scheme was iteratively refined over four rounds of discussion and comparison among the research team. During this process, overlapping or redundant codes were merged, and definitions were clarified to improve intercoder consistency. In total, 54 main codes were generated inductively across the full dataset, with 347 subcodes. After the third iteration, no new codes emerged from the data, indicating that thematic saturation had been reached. The fourth and final round focused on merging the remaining overlapping codes, refining code definitions, and ensuring consistent application of the coding scheme across the entire dataset. All inductively developed codes were subsequently integrated meaningfully into the predetermined deductively established main categories (e.g., Inputs, Activities, Outcomes I–III), confirming the appropriateness of our integrated framework. Supplementary Table S1 illustrates the integration of our deductive theoretical framework with inductively generated codes across all logic model categories.

The first step, familiarisation with the data (Braun & Clarke, 2012), was accomplished through an in-depth reading and re-reading of the interview transcripts, accompanied by note-taking and a discussion of initial impressions. In the second step (generating initial codes), two researchers independently coded the full set of verbatim transcripts according to the deductive framework while remaining open to the emergence of new codes outside the predetermined categories.

During the entire process, the researchers met regularly to resolve discrepancies, refine code definitions, and ensure coding consistency through an iterative process (steps 3–4: searching for and reviewing themes). This process allowed us to systematically map participant perspectives onto the key elements of the programme’s theory and logic while capturing additional themes that emerged inductively from the data (step 5: defining and naming themes).

By combining Braun and Clarke’s structured method with this hybrid deductive–inductive coding technique, we were able to provide a comprehensive analysis that synthesised participant experiences in relation to the intended components and outcomes of the IP, while also remaining sensitive to novel or unexpected insights (step 6: producing the report).

This approach allowed us to leverage existing theoretical frameworks and remain open to the unique aspects of IPs, thereby enhancing both the rigour and richness of our analysis.

Within these broad categories, we then employed an inductive approach, allowing for the emergence of new codes and themes directly from the data. This inductive process enabled us to capture nuanced aspects of IPs and the specific relational dynamics observed in our case study. This hybrid approach aligns with a qualitative research methodology that recognises the value of both theory-driven and data-driven analysis (Fereday & Muir-Cochrane, 2006).

Two researchers independently coded all transcripts using MAXQDA software (VERBI Software, 2020) and met regularly to discuss them.

### Quality Assurance

To ensure data quality and trustworthiness, we implemented several measures:

1. Use of trained interviewers and detailed interview protocols.
2. Verbatim transcription by an independent transcriptionist.
3. Independent coding by two researchers.
4. Regular team meetings for code refinement and consistency checks.
5. Intercoder reliability check on a random selection of transcript segments.
6. Constant comparative method throughout the analysis.
7. Peer debriefing with the entire research team.

## Results

**Fig. 1:**
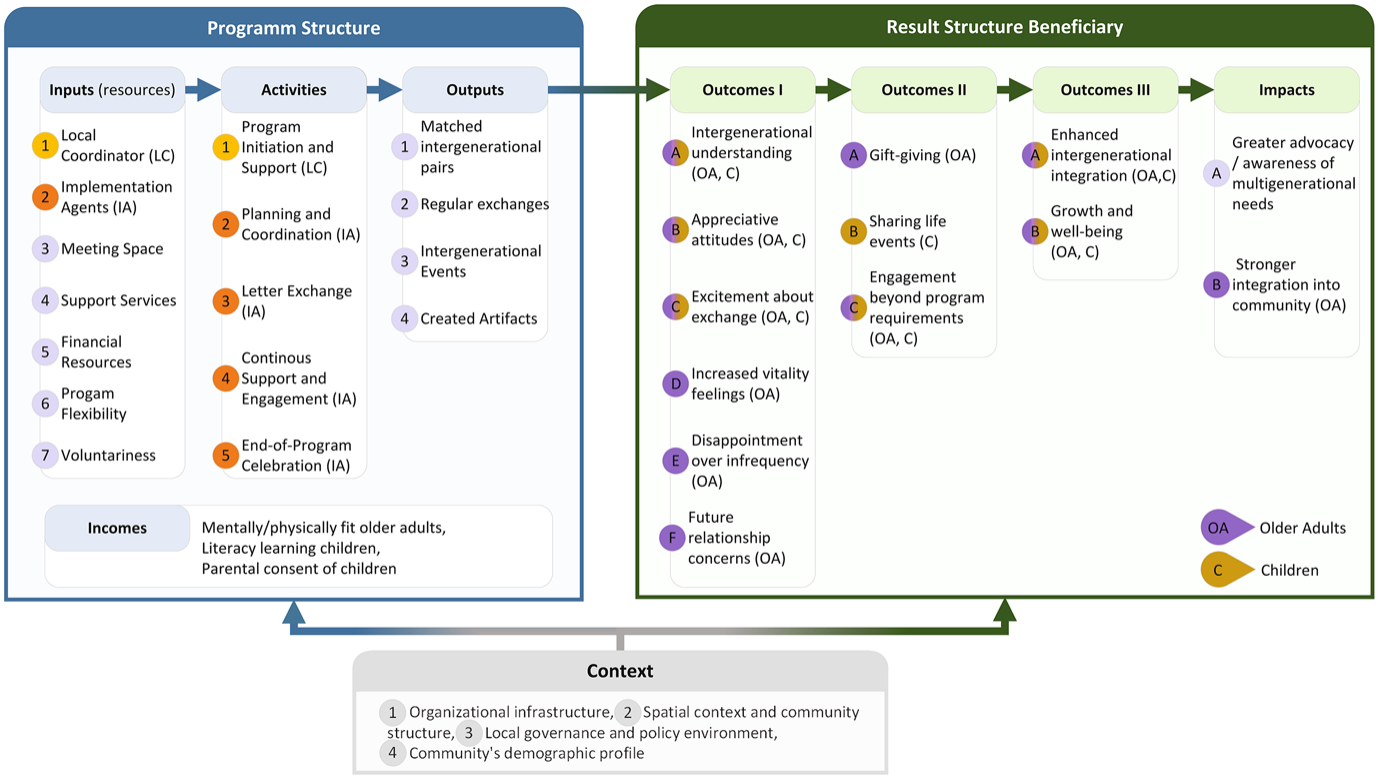
Logic model framework for community-dwelling, non-familial intergenerational programs. The framework illustrates how programme structures (Inputs, Activities, Outputs) lead to individual-level outcomes (Outcomes I–III) and system-level impacts within a specific context. Numbered elements (1–7, A–F) correspond to detailed descriptions in the Results section. OA older adults, C children, LC local coordinator, IA implementation agents

The analysis of the ‘Post für Dich!’ [Mail for You] IP yielded rich insights that contributed to the development of a comprehensive logic model framework for community-dwelling, non-familial IPs. Figure 1 provides a visual representation of this framework, illustrating how programme structures (Inputs, Activities, Outputs) lead to positive and negative Outcome I, Outcome II and Outcome III at the individual level and to impacts at the system level. The following results correspond to the numbered elements in Fig. 1.

The logic model framework consists of several interconnected elements: Inputs, Incomes, Activities, Outputs, Outcomes I–III, Impacts, and Context (see Table 2 for a general description of these elements). Each of these components plays a crucial role in the successful implementation and effectiveness of IPs.

### Inputs

Seven essential inputs emerged from our analysis as critical resources for implementing effective IPs. First, local coordinators (1) serve as crucial community connectors who understand local stakeholders and their needs, acting as pivotal facilitators for programme initiation while promoting social inclusion and intergenerational knowledge exchange. Second, implementation agents (2) from each beneficiary group setting function as committed change agents (also known as change agents in some literature, including World Health Organisation [WHO] guidelines) that enable organisational readiness and provide direct access to target populations, with responsibility for facilitating, overseeing, and delivering core programmatic components to beneficiaries as intended (Pfadenhauer et al., 2017). Third, accessible meeting spaces (3) must be secured within the community to provide available and welcoming social venues for meaningful intergenerational gatherings. Fourth, comprehensive support services (4) should be arranged, including essential logistical assistance, such as transportation for older adults and guidance with technology use. Fifth, adequate financial resources (5) must be secured to support programme management personnel, essential services, and core programme activities. Sixth, programme flexibility (6) should be deliberately incorporated, allowing for modification of activities based on participants’ evolving preferences and emerging needs. Finally, voluntariness (7), particularly regarding older adult participation, represents a fundamental principle ensuring autonomous engagement and respecting self-determination of the participants.

### Incomes

Effective IPs should consider specific characteristics when selecting beneficiary groups, as two key populations emerged from our analysis. For older adults, participants should be members of existing organisational settings (e.g., living facilities for older adults) where they can be easily approached, demonstrate openness to new relationships with children, and maintain physical and mental fitness for activities such as letter writing, reading aloud, and participating in outings. Older adults bring diverse motivations to these programmes, as illustrated by participating older adults: ‘I was simply interested in being around very young people again. And I didn’t feel old at all—in fact, quite the opposite (laughs). I felt a bit lively and young.’ (Older adults, Interviewee 8); ‘But keeping mentally fit—that’s up to me. And I truly believe it’s very, very important. It also brings so much joy that… that the project simply does the soul good.’ (Older adults, Interviewee 6). For children, parental consent must be obtained, and participants should be members of existing organisational settings at developmental ages suitable for acquiring relevant skills (e.g., language and literacy).

### Activities

Five key activities emerged from our interviews and document analyses as essential for successful IP implementation.

First, programme initiation and support (1) requires the local coordinator to assess whether implementation prerequisites exist within the municipality, including organisational settings for both older adults and children and suitable implementation agents from these settings. Necessary conditions, such as municipal administrative support, support services, and financial backing, are then addressed. During this phase, the local coordinator informs potential change agents about programme objectives and their roles, establishes contact with the municipal decision-maker (mayor), submits funding applications to potential agencies, and engages with sponsors; implementation agents begin their work after the local coordinator secures necessary requirements.

Second, planning and coordination (2) involves implementation agents conducting preparatory activities across four domains: identifying and introducing participants through agent-led selection processes and information sessions that emphasise programme benefits, facilitating participant pairing through matching processes that create pairs with common interests or backgrounds, developing scheduling frameworks for letter exchange frequency and overall programme duration, and managing consent and privacy through distribution and collection of consent forms for parents and seniors while communicating privacy policies to ensure confidentiality and safety.

Third, letter exchange (3) involves implementation agents from the children’s settings guiding introductory letter writing with structured templates for sharing interests and asking questions, while agents from older-adult settings assist in crafting age-appropriate responses. Children have expressed enjoyment and fulfilment with this process. As one child noted, ‘I think having a pen pal is really cool, because with phones everything happens so fast, but with letters, it takes longer—and that makes it exciting when one finally arrives’. (Focus group, Children). Another shared with pride, ‘My pen pal even has a folder where she keeps all the letters I’ve written to her so far—and it’s already really big’. (Focus group, Children).

Fourth, continuous support and engagement (4) requires implementation agents to actively promote sustained participation among beneficiaries, with interviews revealing varied motivations among agents. As the mayor explained, ‘The motivation was that we didn’t want to send seniors into well-maintained isolation, but rather ensure they remain an integrated part of our society—actively participating and contributing’. (Interview, Mayor). The teacher emphasised a different focus: ‘For me, it was really more about the children enjoying the process of writing.’ (Interview, Teacher).

Finally, the end-of-programme celebration (5) involves implementation agents organising closing events where beneficiaries, relatives, supporters, and community stakeholders meet, share experiences, and celebrate programme conclusion.

### Outputs

Four essential outputs emerged from our document analysis and project manager interview as key deliverables that effective IPs should aim to produce. First, matched intergenerational pairs (1) based on shared interests create meaningful connections between participants. Second, regular exchanges (2), such as letters and video messages, facilitate ongoing communication between participants throughout the programme duration. Third, multiple intergenerational events (3) provide structured opportunities for face-to-face interactions and relationship building. Finally, tangible artefacts (4) created by participants, including artwork, memoirs, and advice letters, serve as lasting evidence of intergenerational collaboration and programme impact.

## Results

Based on interviews with the older adults and the focus group, IPs should strive for positive results across three progressive levels while remaining aware of potential negative outcomes. These levels build progressively, with each serving as a prerequisite for the next, underscoring the importance of achieving early outcomes to facilitate more substantive changes over time.

**Outcomes I**: Four positive outcomes emerge at this foundational level. First, increased intergenerational understanding (A) develops as children gain knowledge about older adults’ personal histories and past experiences, while older adults learn about contemporary children’s interests and perspectives. Second, appreciative attitudes towards other generations (B) evolve, including recognition of older adults’ positive qualities such as kindness and friendliness, discovery of shared interests and older adults’ sense of humour, and appreciation of children’s honesty and liveliness, with children’s statements reflecting emotional connections built with pen pals. As one child shared, ‘I really like my pen pal because she’s good at baking. (…) When she was young, she wanted to become a baker. And she really likes animals, just like I do’. Another said, ‘I love my pen pal a lot—she’s really funny and nice (…). She always wanted to be a hairdresser when she was younger.’ (Focus group, Children). Third, excitement about intergenerational exchange (D) emerges as participants develop enthusiasm for meaningful interactions across generations, potentially contrasting with typical age-segregated experiences, with one child sharing excitedly: ‘My pen pal is really nice, and I even have her on WhatsApp—she sometimes sends me jokes there. (…) And she can do the splits!’ (Focus group, Children). Fourth, increased feelings of vitality (D) develop among older adults who experience renewed energy and purpose through interactions with children. However, two potential negative outcomes may also emerge at this foundational level. Disappointment over infrequency (E) may develop, as some older adults express dissatisfaction about insufficient meetings and limited opportunities for deep exchanges. Additionally, future relationship concerns (F) may arise as older adults develop fears about relationships ending after the programme conclusion.

**Outcome II**: This second level of outcomes reflects how initial changes in knowledge, attitudes, and skills translate into observable behaviours and actions among participants, as illustrated by one older adult’s comment on her pen pal’s progress: ‘At the beginning, it was funny because he couldn’t spell properly (…). And no one judged him for it, which I think is wonderful. Now he’s in fourth grade and already writes really nice letters.’ (Older adults, Interviewee 4). Three key behavioural changes emerge within this level. First, spontaneous gift-giving between generations (A) develops, including older adults baking for children or providing toys such as soccer balls, or monetary gifts. Second, sharing of life events across generations (B) occurs as participants proactively inform each other about ongoing life developments, demonstrating increased trust and connection, with children sharing significant events and decisions, such as bike tests and school choices, allowing older adults to become part of their lives. Third, spontaneous engagement beyond programme requirements (C) emerges as participants initiate additional interactions outside structured activities, including older adults conversing with parents, family introductions, reciprocal visits between older adults and children with their mothers, joint museum visits, and older adults attending children’s birthday parties.

**Outcomes III** represents significant changes in the life situation and status of beneficiaries, categorised into two main areas: enhanced intergenerational integration (A) and personal growth and well-being (B). Enhanced intergenerational integration (A) encompasses five key developments:

1. Expanded social networks for older adults who experience new relationships, particularly with children, as a primary programme outcome.
2. Extension of intergenerational connections beyond primary participants as children introduce their intergenerational friends to family members, creating ripple effects of cross-generational connections in the broader community.
3. Development of sustained intergenerational relationships where pairs or groups of beneficiaries continue meeting and communicating regularly even after formal programme conclusion, forming lasting bonds.
4. Reduction in age-related stereotypes and segregation as beneficiaries demonstrate more nuanced and positive views about other age groups in daily interactions and decision-making, challenging societal ageism.
5. Establishment of cross-generational support systems where children and older adults begin relying on each other for various forms of support, with older adults offering wisdom and life advice, while younger participants assist with technology or physical tasks.

Personal growth and well-being (B) encompasses two primary developments:

1. Enhanced sense of purpose and enriched daily life for older adults, as ongoing intergenerational connections provide new meaning and variety in daily life experiences.
2. Exposure to diverse life perspectives for children, who gain insights into different historical periods, career paths, and life choices through relationships with older adults, broadening their understanding of life’s possibilities and challenges.

### Impacts

While the Outcomes categories focus on changes at the individual and interpersonal levels, Impacts represent broader effects at the community level encompassing longer-term, far-reaching changes that are the result of successful IP implementation. Two primary impacts emerged from interviews with the mayor and deputy mayor. First, greater advocacy and awareness of multigenerational needs (A) develops as community leaders and policymakers become more attuned to intergenerational initiative benefits, leading to increased support for age-inclusive policies and programmes, with communities experiencing increased cross-generational events and initiatives that foster a more inclusive and connected social fabric. Second, stronger integration of older adults into the community (B) occurs as older adults become more visible and active participants in community events and decision-making processes, breaking down age-based social barriers and potentially leading to increased volunteer participation across age groups, with older adults and youth collaborating on community service projects.

### Context

The context in which an IP operates plays a crucial role in its implementation, outcomes, and broader impacts. Four key contextual factors emerged from our case study as being important for successful IP implementation. First, organisational infrastructure facilitating intergenerational access (1) requires IPs to operate within communities with established organisations where different age groups regularly spend time, such as primary schools and independent senior living communities. These structures provide foundations for organised intergenerational interactions while facilitating easy access to and exchange between beneficiary groups.

Second, spatial context and community structure (2) means IPs operate within defined spatial contexts that facilitate regular interactions between different age groups, characterised by community scales that foster shared identity and cohesion among residents—whether small towns, villages, urban neighbourhoods, or distinct districts within larger cities—combined with geographical proximity between locations where different age groups typically spend time, allowing for easy and frequent intergenerational contact.

Third, local governance and policy environment (3) significantly influence programme implementation and potential for broader impact through local leadership attitudes towards intergenerational initiatives.

Fourth, the community demographic profile (4) requires the presence of different age groups within the community as essential for intergenerational programme implementation.

## Discussion

This qualitative study aimed to develop a comprehensive logic model framework for community-dwelling, non-familial IPs based on a case study of the ‘Post für Dich!’ (‘Mail for You!’) programme in a rural Lower Austrian village. The findings provide valuable insights into the key components, mechanisms, contextual factors, and intended outcomes of such programmes, contributing to the understanding and effective implementation of these interventions.

The framework provides a detailed understanding of how community-dwelling, non-familial IPs function as multi-level interventions. It identifies critical inputs (local coordinator, implementation agents, meeting spaces, and programme flexibility) that align with previous research on effective intergenerational interventions (Hatton-Yeo & Ohsako, 2000; Jarrott et al., 2021). A well-connected local coordinator with knowledge of stakeholders and community needs is pivotal for programme initiation, as recommended by the NICE guideline for older people’s independence and mental well-being (NICE, 2015).

The findings highlight the role of implementation agents in fostering engagement, providing continuous support, and integrating programmatic activities into existing curricula or routines, enhancing sustainability and impact (Hatton-Yeo & Ohsako, 2000). This aligns with the WHO recommendations for effective community engagement using change agents (World Health Organisation, 2020a), a concept similar to the implementation agents described in our framework.

The study underscores the significance of voluntariness for older adult participants, resonating with the principles of autonomy and self-determination in successful ageing initiatives (Rowe & Kahn, 2015). The value of existing organisational settings (e.g., schools and senior living facilities) as avenues for accessing and engaging the target groups echoes previous recommendations for leveraging existing social structures (Kuehne & Melville, 2014).

The identified activities, from programme initiation to intergenerational events, are consistent with good practices for fostering meaningful intergenerational interactions (Jarrott et al., 2021; Kuehne & Melville, 2014). Joint activities beyond letter writing, such as visits, outings, and celebrations, proved crucial for developing new intergenerational relationships.

The described outcomes illustrate the potential for multifaceted benefits of IPs, such as improved attitudes, dispelled age stereotypes, and expanded social networks, aligning with the previous literature (Giraudeau & Bailly, 2019; Murayama et al., 2015; Peters et al., 2021). IPs can contribute to a sense of purpose, usefulness, and enriched daily life for older adults, addressing critical psychosocial needs in later life (Rowe & Kahn, 2015; Steptoe et al., 2015). Recent studies have reinforced these findings, showing that IPs can lead to less depression and better overall well-being among participants (Petersen, 2023).

In terms of impact, IPs have the potential to promote intergenerational awareness and integration within communities, echoing broader societal goals of age-inclusive societies (World Health Organisation, 2020b).

### Implications for Practice and Policy

These findings align with the WHO Age-Friendly Communities framework, which emphasises social participation, respect, inclusion, and civic engagement as essential domains (WHO 2020b). By addressing these domains, IPs offer practical implementation pathways for communities seeking age-friendly designations while addressing social isolation and loneliness among older adults. Such programmes can foster age-inclusive communities, dispel age stereotypes, and promote multigenerational integration, aligning with broader active ageing policy initiatives. IP elements could be incorporated into educational curricula, community centres or senior facilities, leveraging existing resources. However, sustainable implementation across diverse settings requires supportive policies and funding mechanisms.

To foster greater stakeholder engagement and ensure sustained participation in IPs, several strategies could be implemented based on our findings: (1) establish regular feedback mechanisms to monitor participant satisfaction and address concerns promptly, (2) develop recognition systems that acknowledge the contributions of implementation agents and participants, (3) create graduated participation opportunities that allow for varying levels of commitment, and (4) build networks of support among similar programmes to share resources and best practices. These strategies, although not explicitly mentioned by our participants, represent practical applications of the elements identified in our logic model for sustaining long-term engagement.

The logic model framework serves as a practical tool for programme developers, practitioners, and policymakers to design, implement, and evaluate IPs systematically, considering critical inputs, activities, and contextual factors. It can guide programme evaluation efforts by providing a structured approach to assessing inputs, activities, and outcomes, enabling the identification of areas for improvement or refinement. Additionally, it can inform the development of training materials and capacity-building efforts for implementation agents.

The negative outcomes reported by older adults reveal a critical consideration: while IPs aim to reduce isolation, they must be carefully designed to avoid creating new emotional vulnerabilities. Future programmes should include clear information about programme duration, consistent interaction schedules, and strategies for relationship transition or continuation after programme conclusion. These considerations directly address the risk of participants experiencing disappointment or relationship loss, which could undermine the programme’s fundamental goals.

### Strengths and Limitations

A key strength of this study is the theoretically grounded Outcomes categorisation (I, II, and III) and Impact that deliberately aligns with established frameworks. Outcomes I corresponds to Kirkpatrick’s levels 1–2 and the Capability/Motivation components of COM-B; Outcomes II aligns with Kirkpatrick’s level 3 and the behaviour component of COM-B; while Outcomes III reflects Kirkpatrick’s level 4, representing sustained changes on system levels. This theoretical integration enhances both the conceptual robustness and practical utility of the framework.

The use of qualitative methods, such as semi-structured interviews, focus groups, and genuine document analysis, ensures a deep and nuanced understanding of the participants’ perspectives and the programme components of IPs. By incorporating the views of children, older adults, teachers, and community leaders, the study captures a holistic view of IP Outputs, Outcomes, and Impacts, which is crucial for understanding the multi-level benefits and challenges. Notably, the inclusion of children’s perspectives addresses a significant gap in the existing research​​ (Jarrott et al., 2021).

However, the focus on a single case study in a rural Austrian setting may limit the generalizability of the findings to different cultural contexts, urban settings, or communities with different demographic structures and social infrastructures. The reliance on self-reported data may introduce biases, as participants may provide socially desirable responses. The lack of longitudinal data precludes an assessment of long-term outcomes and sustainability. The gender imbalance in the interviews, with predominantly female older adults, may not fully represent the experiences of all older adults involved in the programme. Additionally, the study reports on limited negative outcomes or challenges faced by participants, which may indicate potential bias in data collection or reporting.

### Future Research and Applications

Future research should address several methodological limitations. A mixed methods approach, combining qualitative insights with standardised outcome measures, could address potential self-reporting biases while providing more robust evidence of effectiveness. Comparative studies examining multiple IPs simultaneously could identify which programme elements are most impactful across different contexts. Additional longitudinal designs with follow-up assessments would capture the sustainability of intergenerational relationships beyond the programme conclusion and provide insight into how negative outcomes might evolve over time. Participatory research approaches that more actively involve children in the research process could address the current underrepresentation of their perspectives. This would provide a broader understanding of IP outcomes. Using standardised measures and rigorous methodologies would complement the qualitative findings and contribute to a more comprehensive evidence base. For practical applications beyond the rural Austrian context, the logic model should be adapted by (1) adjusting activities to align with local intergenerational norms and practices, (2) modifying the implementation agent roles to fit existing organisational structures, and (3) refining outcome indicators to reflect culturally specific success measures. Testing in urban settings, where spatial proximity and community cohesion often differ, and in communities with different baseline intergenerational interaction patterns, would significantly strengthen the framework’s versatility and applicability. Such adaptations would address the contextual limitations identified in this single case study while preserving the core structural elements of the model.

## Conclusion

This study makes a significant contribution to the literature by providing an empirically grounded logic model framework for community-dwelling, non-familial IPs. The framework serves as a valuable tool for programme developers, practitioners, and researchers, facilitating the design, implementation, and evaluation of such interventions while considering the complex interplay of inputs, activities, outcomes, and contextual factors.

## Electronic Supplementary Material

Below is the link to the electronic supplementary material.

Supplementary Material 1

## Data Availability

Due to the small community context and distinctive participant roles, the German interview transcripts were pseudonymised but may retain indirect person-related characteristics. In compliance with GDPR, raw transcripts are not publicly available to protect participant privacy. To ensure research reproducibility, the complete German MAXQDA codebook and anonymised coding examples (as shown in Supplementary Table S1) are available from the corresponding author upon reasonable request.
