# Supplementary material for: Developing a Logic Model Framework for Community-Dwelling, Non-Familial Intergenerational Programmes
Source: J Prev (2022). 2025 Dec 6;47(2):353–71. doi: 10.1007/s10935-025-00880-9 (PMC13046647; doi:10.1007/s10935-025-00880-9)
Supplement: Supplementary file 1 — Supplementary Material 1 [file 10935_2025_880_MOESM1_ESM.docx]

Supplementary Table S1

*Coding Structure Overview: Integration of Deductive and Inductive Codes*

Supplementary Table S1 provides representative examples of the coding process, showing how participant perspectives were systematically categorised while maintaining analytical rigour. The numbers in parentheses (n = X) indicate the frequency of coded text segments for each example code; additional codes not shown for brevity. Coding was performed in German (original interview language) to maintain semantic accuracy. Full German MAXQDA codebook available upon request to ensure reproducibility.

| **Deductive Codes** | **Inductive Codes (Examples)** | **Examples: Inductive Subcodes (Examples)** |
| --- | --- | --- |
| Input | Local coordinator | - Project management resources (n = 34) - Interface person (n = 5) - … |
|  | Implementation agents | - Innovative, engaged, organised project leader (n = 16) - Proactive contact initiation (n = 1) - Project implementable without pressure for coordinators (n = 2) - Project lead motivated older adults (n = 2) - Motivation through clear communication (n = 1) - … |
|  | Meeting spaces | - Social spaces for meetings, barrier-free and close to the home of older adults and the school (n = 3) - … |
|  | Support services | - IP provided transport support and financial support for items such as snacks (n = 1) |
|  | Financial resources | - No financial resources needed from the older adults’ side (n = 1) - Training for project leader to develop new ideas (n = 1) - … |
| Incomes | Mentally/physically fit older adults | - Older adults must be willing to step beyond their comfort zones (n = 4) - … |
|  | Parental consent of children | - Family situation did not previously allow for intensive contact with older adults (n = 2) - Parents met older adult at the start of the project to get to know each other (n = 1) - Parents were enthusiastic (n = 1) - Parents should know where their child is going and who they are with (n = 7) |
| Activities | Programme initiation and support | - Project leader motivated participation (n = 14) - … |
|  | Planning and coordination | - Profile exchange as first activity (n = 1) - Pre-selection: Children and older adults brought together based on profile (n = 1) - … |
|  | Letter exchange | - Children wrote letters/postcards (n = 19) - Older adults wrote letters back to child (n = 5 ) - Video message from older adults to children during COVID-19 (n = 2) - … |
|  | End-of-programme celebration | - Older adults visited children's school festival (n = 4) - … |
| Outputs | Matched intergenerational pairs | - Pre-selection: Children and older adults brought together based on profile (n = 1) - Older adult’s wish (girl as pen pal) fulfilled (n = 2) - … |
|  | Regular exchanges | - Children wrote letters/postcards (n = 19) - Older adults wrote letters back to child (n = 5) - Activities/exchange took place approximately every two months (n = 2) - … |
|  | Intergenerational events | - Joint activities and outings (n = 44) - Reading night and joint breakfast (n = 7) - (Hiking) excursion (n = 8) - Joint Mother’s Day breakfast (n = 7) - Older adults visited children's school festival (n = 4) - … |
|  | Created artefacts | - Children designed letters/greeting cards (n = 8) - Children wrote letters with Paint and PowerPoint (n = 1) - Older adult taught child knitting (n = 1) - Joint crafting (n = 3) - … |
| Outcomes I | Intergenerational understanding | - Children gained knowledge about older adults’ experiences (n = 5) - Older adults learned about children’s contemporary interests (n = 8) - Mutual learning and knowledge exchange (n = 12) - … |
|  | Appreciative attitudes | - Children developed positive views of older adults (n = 7) - Older adults appreciated children’s honesty and liveliness (n = 6) - Recognition of shared interests (n = 4) - Discovery of older adults’ humour (n = 3) - … |
|  | Excitement about exchange | - Children expressed enthusiasm for pen pal relationship (n = 9) - Older adults showed excitement about interactions (n = 6) - Both looked forward to meetings and letters (n = 8) - … |
|  | Increased vitality feelings | - Older adults felt energised by children (n = 4) - Renewed sense of purpose (n = 3) - Feeling younger through contact (n = 2) - … |
|  | Disappointment over infrequency | - Older adults wanted more frequent meetings (n = 3) - Wishes for deeper exchanges (n = 2) - … |
|  | Future relationship concerns | - Fears about relationship ending (n = 2) - Uncertainty about continuation (n = 1) - … |
| Outcomes II | Gift-giving | - Older adults gave gifts to children (n=6) - Children gave gifts to older adults (n = 3) - Older adults baked for children (n = 4) - … |
|  | Sharing life events | - Older adults shared personal histories (n = 8) - Children shared school experiences (n = 5) - Informing each other about ongoing life events (n = 6) - … |
|  | Engagement beyond programme requirements | - Visits beyond programme activities (n = 12) - Introduction to family members (n = 7) - Joint activities initiated by participants (n = 9) - Spontaneous visits outside project activities (n = 2) - Children and mothers visit older adults (n = 3) - … |
| Outcomes III | Enhanced intergenerational integration | - Expanded social networks for older adults (n = 8) - Development of sustained relationships (n = 6) - Reduction in age-related stereotypes (n = 4) - Cross-generational support systems (n = 3) - … |
|  | Growth and well-being | - Enhanced sense of purpose for older adults (n = 5) - Enriched daily life experiences (n = 7) - Exposure to diverse life perspectives for children (n = 4) - Broadened understanding of life possibilities (n = 3) - … |
| Impacts | Greater advocacy/awareness of multigenerational needs | - Community leaders more attuned to intergenerational benefits (n = 2) - Increased support for age-inclusive policies (n = 1) - More cross-generational community events (n = 1) - … |
|  | Stronger integration into community | - Older adults more visible in community events (n = 3) - Increased volunteer participation across ages (n = 2) - Breaking down age-based social barriers (n = 1) - Community would support such a project again (n = 1) - … |

n = number of coded text segments;... = additional codes not shown for brevity.
